# An optimized dissociation protocol for FACS-based isolation of rare cell types from *Caenorhabditis elegans* L1 larvae

**DOI:** 10.1016/j.mex.2020.100922

**Published:** 2020-05-16

**Authors:** Euclides E. Fernandes Póvoa, Annabel L.P. Ebbing, Marco C. Betist, Christa van der Veen, Hendrik C. Korswagen

**Affiliations:** a Hubrecht Institute, Royal Netherlands of Arts and Sciences and University Medical Center Utrecht, Uppsalalaan 8, 3584 CT Utrecht, Netherlands; b Institute of Biodynamics and Biocomplexity, Developmental Biology, Department of Biology, Utrecht University, Padualaan 8, 3584 CH Utrecht, Netherlands

**Keywords:** *C. elegans*, L1 larvae dissociation, Single cell isolation

## Abstract

Single-cell isolation and transcriptomic analysis of a specific cell type or tissue offers the possibility of studying cell function and heterogeneity in time-dependent processes with remarkable resolution. The reduced tissue complexity and highly stereotyped development of *Caenorhabditis elegans*, combined with an extensive genetic toolbox and the ease of growing large tightly synchronized populations makes it an exceptional model organism for the application of such approaches. However, the difficulty to dissociate and isolate single cells from larval stages has been a major constraint to this kind of studies. Here, we describe an improved protocol for dissociation and preparation of single cell suspensions from developmentally synchronized populations of *C. elegans* L1 larvae. Our protocol has been empirically optimized to allow efficient FACS-based purification of large number of single cells from rare cell types, for subsequent extraction and sequencing of their mRNA.

## Graphical abstract

**Figure:**
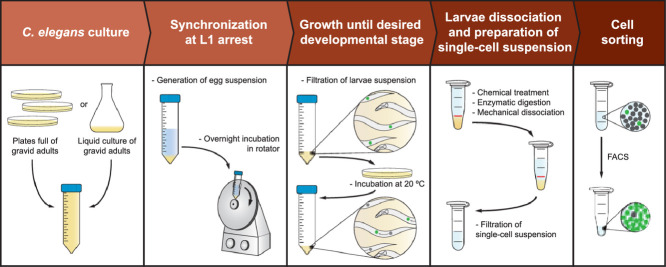

Specifications table

**Table:** 

Subject Area	Biochemistry, Genetics and Molecular Biology
More specific subject area	*C. elegans* developmental biology
Protocol name	Cell isolation of *C. elegans* L1 larvae
Reagents/tools	■ 90 mm Nematode Growth Media (NGM) agar plates ■ NA22 bacteria ■ M9 buffer ■ 5 M NaOH ■ Fresh sodium hypochlorite (household bleach) ■ SDS-DTT solution (*) ■ 20 mg/mL Pronase E solution (*) ■ Egg buffer (*) ■ L-15/FBS medium (*) ■ 50 mL conical centrifuge tubes ■ Glass funnel ■ 20 µm nylon filter (Sigma, NY2004700) ■ 32 mm syringe filter (5 μm pore size) (VWR International, 514–4129) ■ 21G1½ syringe needles (or bigger) (BD Eclipse Needle 21G1½, 305,895) ■ 1 mL syringe (BD Plastipak, 303,172) ■ 1.5 mL microcentrifuge tubes ■ 2 mL microcentrifuge tubes ■ Centrifuge (compatible with 50 mL conical centrifuge tubes) ■ Benchtop microcentrifuge ■ Tube rotator ■ Pellet pestle motor (Sigma, Z359971) ■ Pellet pestles (Sigma, Z359947-100EA)
	(*) See Appendix A: Supplementary material for a full description of the solution preparation.
Experimental design	This protocol consists of four main steps:
	1. *C. elegans* culture preparation and growth 2. Synchronization of larvae at L1 arrest 3. Obtaining synchronized populations of specific developmental stages 4. Larvae dissociation and preparation of single-cell suspension
Trial registration	
Ethics	
Value of the Protocol	■ Generation of single cell suspensions can be achieved in a 2.5 h compared to the 3 h of previous protocols, reducing transcriptional effects due to stress and loss of viable cells when FACS sorting. ■ Efficient isolation for rare cell types. ■ The quality of single cell suspensions allows its use for single cell RNA sequencing.

## Description of protocol

Here, we present an optimized protocol for the isolation of large numbers of rare cell types from *C. elegans* L1 larvae. We built on the knowledge from previous work ([1], [2], [3]), and empirically developed a faster and more robust protocol. This protocol was optimized for the isolation of neuroblasts (Q lineage) as well as epidermal cells (seam cells), but it has also been successfully applied to other cell types such as mesoblasts (M lineage) (Molly Godfrey and Sander van den Heuvel personal communication). Note that even though this protocol was optimized for the isolation of cells from the L1 larval stage, adjustments in the chemical and mechanical treatments steps would in principal make it amenable for the isolation of cells from other larval stages.

To improve the overall output and reduce the time needed for the procedure, we have introduced new steps such as filtration of the L1 arrested larvae sample and filtration of the final cell suspension, as well as the adjustment of the sample washing upon SDS-DTT treatment, which substantially reduces the time of this critical step. In addition, we reformulated the laborious mechanical treatment of the sample by introducing the use of a pellet pestle motor, which allows a more consistent and user-friendly procedure. Finally, we have removed the need for aseptic conditions when growing the *C. elegans* cultures, a change that had no impact on the quality of the cell sample and the downstream processing. This change not only contributes to a more user-friendly procedure, but also reduces its technical requirements. It should be noted, however, that this protocol was optimized for use of the cell suspension for FACS and subsequent RNA isolation. If cell culturing is intended, the aseptic *C. elegans* culturing conditions presented by Zhang and colleagues should be taken into consideration.

1. C. elegans culture preparation and growth

1.1. Seed one hundred 90 mm NA22 plates by chunking approximately 100 worms from an ongoing (non-starved) culture.
  NOTE 1: Use NGM plates seeded with 800–1000 µL NA22 bacteria.
  NOTE 2: Animals to be seeded can be obtained from both synchronous and asynchronous populations. The use of a synchronous population allows maximizing the output of the protocol.
  NOTE 3: In our experience, 80–100 plates ensure a good cell yield even in situations where there is a higher loss of sample during the steps of this protocol or when chemical and mechanical treatments are less efficient.
1.2. Incubate at 20 °C for multiple generations until the plates are full of gravid adults (shortly before the plates reach starvation).
  NOTE: It is important to not let the plates starve completely as this may affect development and gene expression.
  IMPORTANT: C. elegans culture on solid plates can be replaced by growth in liquid cultures. In Appendix B, we present an adapted protocol for such culture. If this method is chosen, ignore steps 1.1 and 1.2.

2. Synchronization of larvae at L1 arrest

2.1. Collect animals with M9 buffer into 50 mL conical centrifuge tubes. If necessary, use multiple tubes.
  NOTE: We typically use 2–3 tubes for this step.
2.2. Centrifuge for 2 min at 800 g.
2.3. Remove the top part of the M9 buffer, leaving the bottom 35 mL.
  NOTE: If there is too much bacteria in suspension, remove most of the M9 buffer (being careful with the pellet), resuspend in M9 buffer, and repeat steps 2.2 and 2.3.
2.4. Add 5 mL 5 M NaOH and 10 mL fresh sodium hypochlorite.
  NOTE: Do not use sodium hypochlorite open for more than two weeks as this reduces the efficiency of the worm dissociation.
2.5. Vortex the sample for 5 min at maximum speed.
  NOTE: Vortex time can be extended in order to decrease the number of carcasses and debris. However, this may result in a decrease in the number of hatching eggs.
2.6. Immediately centrifuge the sample for 2 min at 1300 g.
2.7. Remove most of the supernatant and resuspend the pellet in 50 mL M9 buffer.
2.8. Centrifuge the sample for 1 min at 1300 g.
2.9. Repeat steps 2.7 and 2.8 two more times.
2.10. Resuspend the sample in 25 mL M9 buffer.
2.11. Place the tube in a rotator and incubate overnight (12–18 h).
  NOTE: Incubation times shorter than 12 h will result in a suboptimal number of hatched eggs.
2.12. Place two 20 µm nylon filters into a glass funnel and pour the suspension of overnight hatched larvae into a new 50 mL conical tube.
  NOTE: If a large number of carcasses and debris are present in the suspension this will result in clogging of the filters and consequent loss of a significant part of the sample. In order to minimize the number larvae lost in this step, two approaches can be taken: 1) split the sample into two 50 mL conical tubes, and filter the content of each tube separately; or 2) resuspend the clogged fraction of the sample and repeat step 2.1 using new filters.
  IMPORTANT: If L1 arrested larvae are intended to be used proceed to step 3.6, ignoring steps 3.1–3.5, otherwise proceed with step 3.1.

3. Obtaining synchronized populations of specific developmental stages

3.1. Centrifuge filtered larvae for 1 min at 1300 g.
3.2. Remove most of the supernatant, leaving 2.5–5 mL in the tube.
3.3. Resuspend the pellet by gently pipetting up-and-down and transfer the larvae suspension onto fresh NA22 plates previously incubated at room temperature.
  NOTE 1: Typically, 5 to 10 plates are necessary for this step. This number should be adjusted to the number of larvae present in the sample.
  NOTE 2: It is important to ensure that the larvae are not pipetted onto areas of the plate not covered with bacteria as this will affect the developmental synchrony of the population.
3.4. Incubate at 20 °C until animals have reached the desired developmental stage.
  NOTE: Going through the L1 arrested stage affects the timing of developmental events compared to non-starved populations. We recommend prior optimization to determine the optimal incubation time by performing steps 1.1–3.6 of this protocol followed by an evaluation of the desired developmental stage. Multiple incubation times can be tested with a single batch of L1 arrested larvae.
3.5. After incubation, collect animals with M9 buffer into a 50 mL conical tube.
3.6. Pellet the sample by centrifuging for 1 min at 1300 g.
3.7. Remove most of the supernatant and resuspend the sample in 50 mL M9 buffer.
3.8. Repeat 1–2 times steps 3.6 and 3.7 to remove as much bacteria as possible.
  NOTE: This step is not necessary if L1 arrested worms are being used.
3.9. Remove most of the supernatant, leaving approximately 1.5 mL (including pellet).
  IMPORTANT: At this point, place the SDS-DTT and Pronase E aliquots at room temperature. Before using it, make sure the SDS-DTT solution is clear of precipitates. If this is not the case, warm up the aliquot in the hand and vortex until it is clear.

4. Larvae dissociation and preparation of single-cell suspension

4.1. Prepare a 1.5 mL microcentrifuge tube by filling it with 100 µL of sterile water and marking the water level on the tube. Remove the water.
4.2. Resuspend the larvae pellet and transfer it to the marked 1.5 mL tube.
4.3. Centrifuge for 2 min at 16,000 g to pellet the larvae.
4.4. Carefully remove nearly all the supernatant, leaving the pellet as compact as possible.
  NOTE: If the volume of the compacted larvae pellet exceeds the 100 µL mark, split the sample into multiple 1.5 mL tubes, and repeat steps 4.4 and 4.5. Note that in this case it will be needed to thaw as many SDS-DTT and Pronase E aliquots as the number of 1.5 mL tubes used.
  IMPORTANT: Execute steps 4.5–4.9 as fast as possible, as cell viability decreases if incubated in SDS-DTT solution for more than a couple of minutes.
4.5. Add 500 µL SDS-DTT solution to each tube and gently flick the bottom of the tube until mixed. Incubate for 2 min in the rotator.
4.6. Immediately after the SDS-DTT incubation, add 800 µL egg buffer to the reaction. Flick gently to mix.
4.7. Pellet the larvae by centrifuging at 16,000 g for 15 s.
4.8. Remove the supernatant and add 1 mL egg buffer. Resuspend the pellet by flicking the tube.
4.9. Repeat steps 4.7 and 4.8 at least 4 times.
4.10. Pellet the larvae at 16,000 g for 15 s. Remove supernatant until the 100 µL mark.
4.11. Add 500 µL of 20 mg/mL Pronase E and incubate for 10 min in the rotator.
4.12. Use the pellet pestle motor for 2 min, to dissociate worms and cells by mechanical force.
  NOTE: Be careful as the solution may spill out from the tube during this step.
4.13. Place the tube back on the rotator and let the sample incubate in Pronase E for an additional 30–35 min.
4.14. Add up to 800 µL cold l-15/FBS to stop the reaction.
4.15. Pellet the dissociated cells by centrifuging at 9600 g for 5 min at 4 °C. Remove the supernatant.
4.16. Add 1 mL cold l-15/FBS and resuspend the cell pellet by gently pipetting up-and-down.
4.17. Repeat steps 4.15 and 4.16 two more times. Put the tube on ice.
  IMPORTANT 1: From this moment on, always keep the sample on ice.
  IMPORTANT 2: Place a 2 mL tube on ice before proceeding.
4.18. Assemble a sterile 21G1½ needle onto the 1 mL syringe and take in 1 mL cold l-15/FBS.
4.19. Replace the needle with a 5 µm filter and slowly discharge the content of the syringe by applying constant force. Register the volume at which the solution starts to come out of the filter tip.
4.20. Reassemble the needle on the syringe and take in the 1 mL of cell suspension.
4.21. Replace the needle with the 5 µm filter and slowly push the sample through the filter by applying constant force until the value registered in 3.17.
4.22. Filter the rest of the sample into the 2 mL tube previously placed on ice.
4.23. Assemble a new sterile needle on the syringe and take in 1 mL cold l-15/FBS.
4.24. Replace the needle with the 5 µm filter and slowly push the full 1 mL into the 2 mL tube containing the sample.
4.25. Pellet filtered cells by centrifuging at 9600 g for 5 min at 4 °C.
4.26. Remove the supernatant and resuspend the cells in 1 mL cold l-15/FBS by gently pipetting up-and-down.
4.27. Keep the tube on ice until cell sorting.
  NOTE: To maximize the output of the experiment, it is important to minimize the time between the end of the protocol and the sorting of the cells. We typically FACS sort the cells no more than 30 min after the last step of the protocol.

*Final considerations regarding quality control and FACS optimization*

Efficiency of the protocol and quality of the sample can be assessed by analyzing the cell suspension samples using a hemocytometer and an adequate microscope as described in Zhang et al., 2013. However, the detection of rare and small fluorescent cells using this procedure is very inefficient and hard to implement. We, therefore, recommend using this procedure only at initial trials of the protocol to assess the overall quality of the procedure, i.e., if cell dissociation is occurring as expected. In our hands, the most efficient way of evaluating the presence and quality of rare fluorescent cells is by FACS-based isolation of these cells followed by culture on lectin-coated glass bottom dishes as described in Zhang et al., 2013. Cells should be incubated for 24 to 48 h to allow adhesion to the bottom of the plate before further analysis.

Optimization of the FACS settings is highly dependent on the morphological features of the cell type of interest, quality and spectral features of the fluorescent markers, and the characteristics of the sorting equipment used. It is, therefore, necessarily a trial and error approach, since it is difficult to establish general rules for this procedure. Nevertheless, the following recommendations can contribute to minimize the optimization time:

50- Use *C. elegans* strains carrying stably integrated transgenes with high expression of the fluorescent marker(s).
50- When setting up the gating strategy, keep in mind that *C. elegans* cells are generally small. Flow cytometry facilities are commonly used to develop strategies to sort mammalian cells which are much larger in size.
50- In each trial, sort different sub-populations that could fit the criteria for the desired cells separately. This can decrease the number of trials needed to find the correct population of cells.
50- In parallel to the evaluation based on the cell culture mentioned above, a fraction of the sample can be resorted to access its purity and efficiency of the sorting.

## Declaration of Competing Interest

The authors declare that they have no known competing financial interests or personal relationships that could have appeared to influence the work reported in this paper.

## Appendix Supplementary materials

Image, application 1

Image, application 2
