# Supplementary material for: An optimized dissociation protocol for FACS-based isolation of rare cell types from Caenorhabditis elegans L1 larvae
Source: MethodsX. 2020 May 16;7:100922. doi: 10.1016/j.mex.2020.100922 (PMC7265044; doi:10.1016/j.mex.2020.100922)
Supplement: Supplementary file 1 [file mmc1.docx]

Appendix A: Supplementary materials

**Egg Buffer**

*Reagents*

- 2M NaCl
- 1M HEPES, pH 7.3 (store at 4 °C)
- 2M KCl
- 1M CaCl_2_
- 1M MgCl_2_

*Preparation*

1. Combine the following reagents at room temperature:

| **Reagent** | **Volume** | **Final concentration** |
| --- | --- | --- |
| NaCl | 29.5 mL | 118 mM |
| HEPES pH 7.3 | 12.5 mL | 25 mM |
| KCl | 12.0 mL | 48 mM |
| CaCl_2_ | 1.0 mL | 2 mM |
| MgCl_2_ | 1.0 mL | 2 mM |
| ddH_2_O | 444.0 mL |  |
| **Total** | **500.0 mL** |  |

1. Before proceeding with the measurements, calibrate the osmometer.
2. Measure the osmolarity. If necessary, adjust osmolarity to 340±5 mOsm by diluting with sterile ddH_2_O.
3. In a tissue culture hood, sterilize the buffer with a 0.22 μm vacuum filter unit.
4. Aliquot the egg buffer in 50 mL tubes to avoid contamination.
5. Store at 4 ºC.

**L-15/FBS medium**

*Reagents*

- Leibovitz's L-15 medium, no phenol red (Invitrogen 21083-027)
- Fetal Bovine Serum (FBS, Invitrogen 16000-077)

*NOTE:* To avoid freezing and thawing of FBS, sterilely aliquot a new bottle into 50 mL tubes.

- Penicillin-Streptomycin solution (Sigma P4458)

*NOTE:* 5,000 units penicillin and 5 mg streptomycin/mL

- 60% w/v Sucrose, in ddH2O (sterile)

*Preparation*

1. Equilibrate the osmometer to room temperature overnight.
2. Thaw FBS overnight at 4 ºC.
3. In a cell culture hood, combine the following reagents sterilely:

| **Reagent** | **Volume** |
| --- | --- |
| L-15 insect media | 450.00 mL |
| 10% FBS | 50.00 mL |
| Penicillin-Streptomycin solution | 5.00 mL |
| **Total** | **505.00 mL** |

1. Before proceeding with the measurements, calibrate the osmometer.
2. Measure the osmolarity. If necessary, adjust osmolarity to 340±5 mOsm by diluting with sterile 60% sucrose.
3. In a tissue culture hood, sterilize the buffer with a 0.22 μm vacuum filter unit.
4. Aliquot the L-15/FBS medium in 15 mL tubes to avoid contamination.
5. Store at 4 ºC.

**Pronase E solution**

*Reagents*

- Pronase E (Sigma P8811)
- Egg buffer

*Preparation*

1. In a tissue culture hood, dissolve Pronase E to 20 mg/mL in cold sterile egg buffer.
2. Vortex thoroughly until dissolved.
3. Sterilely split the solution into 500 μL aliquots.
4. Store at -20 ºC.

**SDS-DTT solution**

*Reagents*

- 1M HEPES, pH 8 (store at 4 °C)
- 10% (w/v) Sodium dodecyl sulfate (SDS, VWR International ICNA04811030) (store at 4 °C)
- 1 M Dithiothreitol (DTT, Sigma 43816) (store at -20 °C)
- 60% (w/v) Sucrose

*Preparation*

1. Combine the following reagents at room temperature:

| **Reagent** | **Volume** | **Final concentration** |
| --- | --- | --- |
| HEPES pH 8.0 | 0.40 mL | 20 mM |
| SDS | 0.50 mL | 0.25% |
| DTT | 4.00 mL | 200 mM |
| Sucrose | 1.00 mL | 3% |
| ddH_2_O | 14.60 mL | 2 mM |
| **Total** | **20.50 mL** |  |

1. In a tissue culture hood, sterilize the solution using a 0.2 μm syringe filter.
2. Split the solution into 500 μL aliquots.
3. Store at -20 ºC.
