# Supplementary material for: An optimized dissociation protocol for FACS-based isolation of rare cell types from Caenorhabditis elegans L1 larvae
Source: MethodsX. 2020 May 16;7:100922. doi: 10.1016/j.mex.2020.100922 (PMC7265044; doi:10.1016/j.mex.2020.100922)
Supplement: Supplementary file 2 [file mmc2.docx]

Appendix B: *C. elegans* liquid culture

*C. elegans worm preparation and growth for liquid culture*

- 1. Seed ten 90 mm HB101 plates and put 15 L4 worms per plate.

*NOTE:* Use NGM plates seeded with 700 μL HB101 bacteria, spread out well over the plate as HB101 bacteria are very viscous.

- 1. Incubate at 20°C until the plates are full of gravid adults.

*Growth of HB101 bacteria for liquid culture*

- 1. Inoculate 4 Erlenmeyer with 600 mL sterile Terrific broth plus 10 mM MgSO_4_ with HB101 bacteria derived from a clean streak and grow for 18 hours at 37°C and 200 rpm.

*NOTE 1:* Growth of HB101 in Terrific Broth increases yield almost two-fold compared to growth in LB at the same growth conditions. Addition of 10 mM MgSO_4_ was found to be optimal to increase bacterial yield.

*NOTE 2:* Use sterilized Erlenmeyer and work sterile in preparation of the bacterial cultures.

- 1. Spin down bacterial cultures in 1 L sterilized buckets for 10 minutes at >3,500g at 4°C.
  2. Pool all bacteria in 100 ml freshly prepared S Medium and store at 4°C until use.

*NOTE 1:* HB101 bacteria are preferably prepared the day before the liquid culture is started, to avoid storage for extended periods. If necessary, the pellets without addition of S Medium can be stored at -20°C.
*NOTE 2:* 400 mL S Medium should be prepared fresh right before the start of the liquid culture.

*Preparation and start of liquid culture*

- 1. Collect animals with M9 buffer into a 50mL conical centrifuge tube.
  2. Centrifuge for 2 minutes at 800g and remove most of the M9 without disturbing the worm pellet.
  3. Wash the worm pellet with fresh M9 once, resuspending the worm in 35 mL M9.
  4. Add 5 ml 5M NaOH and 10 ml fresh sodium hypochlorite.

*NOTE:* Do not use sodium hypochlorite open for more than two weeks as this reduces the efficiency of the worm dissociation.

- 1. 3.5. Vortex the sample for 5 min at maximum speed, immediately followed by centrifugation for 2 minutes at 1,300g.
  2. Remove the supernatant without disturbing the worm pellet.
  3. Add fresh M9 and centrifugate for 2 minutes at 1,300g.
  4. Repeat step 3.6 and 3.7 twice, not adding M9 after the last washing step.
  5. Resuspend the embryos in 50 mL freshly prepared S Medium.
  6. Add 250 mL S Medium to a sterilized 2 L plastic Erlenmeyer.
  7. Add the HB101 bacteria in S Medium to the Erlenmeyer, followed by the embryo suspension in 50 mL S Medium.
  8. Grown cultures in shake incubators at 20°C at 175 rpm for 4 days (adjust to 1 generation depending on strain).

*NOTE:* Prior to continuation to the next step, take a sample (while maintain sterility of the culture) to check for the presence of gravid adults or contaminations.

- 1. Spin down the worm cultures in 200 mL buckets for 3 minutes at 450g and 4°C. Place the buckets on ice for 5 minutes after centrifugation to ensure worms settling to the bottom.
  2. Remove supernatant and transfer the worms to a 50 mL conical tube.
  3. Wash the pellet with M9 until most bacteria are cleared from the worm suspension.
  4. Continue with ‘2. Synchronization of larvae at L1 arrest’ section from the main protocol.

| **Reagent** | **Volume** | **Preparation** | **Manufacturer** |
| --- | --- | --- | --- |
| Terrific Broth | 1L | 47.6 g Terrific Broth powder 4 mL glycerol | VWR (J869)  Boom (76050772) |
| 1M K_2_HPO_4_ | 1L | 174.2 g K_2_HPO_4_  H_2_O to 1 liter  Autoclave | Merck (105104) |
| 1M KH_2_PO_4_ | 1L | 136.09 g KH_2_PO_4_  H_2_O to 1 liter  Autoclave | Boom (76020922) |
| 1M MgSO_4_ | 1L | 120.37 g MgSO_4_  H_2_O to 1 liter  Autoclave | Acros Organics (213115000) |
| 1M CaCl_2_ | 1L | 110.98 g CaCl_2_  H_2_O to 1 liter  Autoclave | Sigma-Aldrich (C2661) |
| Cholesterol | 100 mL | 500 mg cholesterol  Ethanol to 100 mL | Sigma-Aldrich (C3045) |
| Potassium phosphate | 1L | 132 mL 1M K_2_HPO_4_*3H_2_O (228.23 g/L) 868 mL 1M KH_2_PO_4_  Adjust to pH 6.0 by changing balance between solutions |  |
| Trace Metals solution | 1L | 1.86 g disodium EDTA   - 1. g FeSO_4_*7H_2_O   2. g MnCl_2_*4H_2_O   3. g ZnSO_4_*7H_2_O   0.025 g CuSO_4_*5H_2_O  H_2_O to 1 liter  Autoclave and store in the dark | Sigma-Aldrich (E4884)  Sigma-Aldrich (F8633)  Sigma-Aldrich (M3634)  Sigma-Aldrich (Z0251)  Sigma-Aldrich (C8027) |
| Pen/Strep |  | 5000 Units/mL penicillin  5000 µg/mL of streptomycin | Gibco (15070-063) |
| Nystatin |  | \|  \| 10,000 Unit/mL in DPBS \| \| --- \| --- \| | Merck (N1638) |
| S Basal | 1L | 5.844 g NaCl  Autoclave solution | Boom (76028327) |
| S Medium | 1L | 10 mL 1M potassium citrate pH 6.0  10 mL Trace Metal solution  3 mL CaCl_2_  3 mL MgSO_4_  PenStrep (to a final concentration of 200 μg/mL) Nystatin (to a final concentration of 10 μg/mL)  S Basal to 1 liter  Add all reagents using sterile technique, do not autoclave |  |
